# Determinação social da saúde, complexidade, colonialidade e longa duração

**DOI:** 10.1590/0102-311XPT035724

**Published:** 2025-02-07

**Authors:** Gil Sevalho

**Affiliations:** 1 Escola Nacional de Saúde Pública Sergio Arouca, Fundação Oswaldo Cruz, Rio de Janeiro, Brasil.

**Keywords:** Determinação Social da Saúde, Decolonialidade, Epistemologia, Social Determination of Health, Decoloniality, Epistemology, Determinación Social de la Salud, Decolonialidad, Epistemología

## Abstract

Este ensaio relaciona conhecimento crítico da determinação social da saúde, colonialidade e tempo como fundamento epistêmico. O tempo é um elemento relevante de articulação interdisciplinar complexa, embora não conforme reflexão aprofundada na Saúde Coletiva. Resgatam-se bases históricas, e atualiza-se o debate sobre a determinação social da saúde, relevando-se as contribuições de Naomar de Almeida Filho e Jaime Breilh, autores que pensam desde o Sul, fora da hegemonia norte-americana e eurocêntrica. Complexidade, compromisso com a transformação social e sentido descolonizador inspiram a aproximação entre a saúde coletiva, as reflexões de Fernand Braudel e Immanuel Wallerstein, sobre a longa duração e o capitalismo histórico no sistema moderno, e o pensamento decolonial com seu conceito de colonialidade. A concepção braudeliana compreende temporalidades múltiplas e integra, dialeticamente, a mudança e a permanência na longa duração, e a reflexão decolonial insere a colonialidade na longa duração. A colonialidade compõe a determinação social da saúde, ou como dispositivo que produz e reproduz iniquidades econômicas, de gênero, étnico-raciais e opressão social, ou na dimensão epistemológica como colonialidade do saber que orienta a conceituação epidemiológica. A longa duração de Braudel, o sistema-mundo de Wallerstein e o pensamento decolonial são aportes a serem explorados no pensamento em saúde. A integração epistemológica do tempo à reflexão epidemiológica pode revelar apagamentos e (re)constituir contextos históricos e durações cuja complexidade foge à visão fragmentada e congelada dos determinantes produzida pelo funcionalismo cartesiano da epidemiologia dos fatores de risco.

## Abstract (en)

This essay connects critical knowledge on the social determination of health, coloniality, and time as an epistemic foundation. Time is a relevant element in the articulation of complex interdisciplinary work, though it has not been deeply reflected upon in Public Health. The essay revisits historical bases and updates the debate on the social determination of health, highlighting the contributions of Naomar de Almeida Filho and Jaime Breilh - authors whose thoughts primary focus are on the Global South, outside of the North American and Eurocentric hegemony. Complexity, commitment to social transformation, and a decolonizing perspective inspire the intersection between public health, the reflections of Fernand Braudel and Immanuel Wallerstein on the long term and historical capitalism in the modern world-system, and decolonial thought with its concept of coloniality. The Braudelian conception encompasses multiple temporalities and dialectically integrates change and permanence in the long term, while decolonial reflection inserts coloniality into this longue durée. Coloniality is part of the social determination of health, either as a mechanism that produces and reproduces economic, gender, and ethno-racial inequalities and social oppression, or in its epistemological dimension as the coloniality of knowledge behind epidemiological conceptualization. Braudel’s longue durée, Wallerstein’s world-system, and decolonial thought are contributions to be further explored within health thinking. The epistemological integration of time into epidemiological reflection can reveal erasures and (re)constitute historical contexts and durations whose complexity escapes the fragmented and static view of determinants produced by the Cartesian functionalism of risk factor epidemiology.

## Abstract (es)

El ensayo relaciona el conocimiento crítico de la determinación social de la salud, la colonialidad y el tiempo como fundamento epistémico. El tiempo es un elemento relevante de articulación interdisciplinaria compleja, aunque no según una reflexión profunda en Salud Colectiva. Se rescatan bases históricas y se actualiza el debate sobre la determinación social de la salud, destacando los aportes de Naomar de Almeida Filho y Jaime Breilh, autores que piensan desde el Sur, fuera de la hegemonía norteamericana y eurocéntrica. La complejidad, el compromiso con la transformación social, el sentido descolonizador inspiran el acercamiento entre la salud colectiva, las reflexiones de Fernand Braudel e Immanuel Wallerstein sobre larga duración y capitalismo histórico en el sistema mundo moderno, y el pensamiento decolonial con su concepto de colonialidad. La concepción braudeliana comprende múltiples temporalidades e integra dialécticamente el cambio y la permanencia en la larga duración, y la reflexión decolonial inserta la colonialidad en la larga duración. La colonialidad conforma la determinación social de la salud, ya sea como un dispositivo que produce y reproduce las desigualdades económicas, de género, étnico-raciales y la opresión social, o en la dimensión epistemológica como la colonialidad del saber que orienta la conceptualización epidemiológica. La larga duración de Braudel, el sistema-mundo de Wallerstein y el pensamiento decolonial son aportes que deben explorarse en el pensamiento en salud. La integración epistemológica del tiempo en la reflexión epidemiológica puede revelar desvanecimientos y (re)constituir contextos y duraciones cuya complejidad escapa a la visión fragmentada y congelada de los determinantes producida por el funcionalismo cartesiano de la epidemiología de los factores de riesgo.

## Introdução

Acompanhando as mudanças sociodemográficas mundiais e seu impacto na saúde, o olhar epidemiológico passou dos modos de transmissão das doenças infecciosas para uma epidemiologia dos fatores de risco, abrangendo então agravos à saúde e doenças não infecciosas. O conhecimento da saúde de populações evoluiu de uma visão unicausal para uma compreensão multicausal, e, posteriormente, voltou sua atenção para o estudo da determinação social da saúde no contexto da epidemiologia dos fatores de risco.

Para Buss & Pellegrini Filho ^1^ (p. 80), “*apesar da preponderância do enfoque médico biológico na conformação inicial da saúde pública como campo científico*”, permanece a tensão entre essa visão e a relevância dada aos fatores ambientais e sociopolíticos no processo saúde-doença-cuidado.

Avaliar dimensões sociais e biológicas do processo saúde-doença-cuidado é questão fundamental para a Epidemiologia, e a ênfase no social é central na construção epistêmica crítica da Saúde Coletiva. A determinação social da saúde, sobretudo, tornou-se tema conceitual aberto ao debate, e inscreve-se na discussão a definição produzida pela Organização Mundial da Saúde (OMS) em meados do século XX, que descreve a saúde como o estado de completo bem-estar físico, mental e social e não somente a ausência de doença.

Conceituada assim, a saúde tem dimensões sociais além da ordem biológica, mas observe-se o apontamento de Almeida Filho ^2^ (p. 6) de que a Epidemiologia não é capaz de produzir uma “*referência teórica eficaz sobre o objeto saúde*”, tanto mais diante do “*todo completo*” idealizado pela OMS. Para Almeida Filho, são comuns às disciplinas da área da saúde a ênfase no individual e uma insistência em construir conceitualmente a doença, o patológico. A Epidemiologia alcança uma coletivização da doença por meio do conceito de morbidade, mas tanto ela como a clínica fracassam em afirmar a saúde. Segundo Almeida Filho ^2^ (p. 7), saúde constitui “*ponto cego*” da Epidemiologia, em função da consideração “*parcial e residual*” da “*doença coletiva*” por meio do “*risco e seus fatores*”.

Pretendemos explorar relações entre o conhecimento crítico da determinação social da saúde, a colonialidade e o tempo como fundamento epistêmico. Nessa perspectiva, associamos o capitalismo histórico com a produção de iniquidades, entre as quais salientamos as étnico-raciais. Resgatamos bases históricas e situamos o debate contemporâneo sobre a determinação social da saúde, destacando as reflexões de Naomar de Almeida Filho e Jaime Breilh, autores expressivos no contexto latino-americano, enfatizando suas abordagens da pandemia de COVID-19. Privilegiando as orientações epistêmicas da complexidade e o questionamento sobre a colonização do saber, conjugamos a determinação social da saúde com a longa duração, o sistema-mundo e a colonialidade, tendo como referências Fernand Braudel, Immanuel Wallerstein e o pensamento decolonial.

A longa duração aparece no discurso decolonial, e a colonialidade é essencial nessa integração. O tempo é elemento de articulação epistemológica que alimenta a interdisciplinaridade e a complexidade, embora essa perspectiva não constitua reflexão aprofundada na Saúde Coletiva.

Este texto é um ensaio, forma que procura explorar e desvendar em vez de descrever, cuja natureza crítica interroga sobre o que o controle do saber oculta e a objetividade impõe ^3^.

## Os determinantes sociais da saúde

Para Buss & Pellegrini Filho ^1^ (p. 78), “*as definições de determinantes sociais da saúde*” consideram a relação entre condições de vida e trabalho e a saúde de indivíduos e populações. Essa compreensão ganhou dimensão oficial internacional com a criação de instâncias organizativas vinculadas à OMS. Para a Comissão Nacional sobre Determinantes Sociais da Saúde, criada no Brasil em 2006, determinantes sociais de saúde “*são os fatores sociais, econômicos, culturais, étnicos/raciais, psicológicos e comportamentais que influenciam a ocorrência de problemas de saúde e seus fatores de risco na população*” ^1^ (p. 78). Criada em 2005, a comissão homônima da OMS postula que os determinantes sociais da saúde “*são as condições sociais em que as pessoas vivem e trabalham*” ^1^ (p. 78).

O estudo da determinação social da saúde tem produzido teorias explicativas, das quais destaca-se o consagrado modelo idealizado por Dahlgren & Whitehead ^4^. O modelo tem forte influência na compreensão da questão e na normatização de recomendações estabelecidas pela OMS.

Na descrição de Buss & Pellegrini Filho ^1^, o modelo apresenta os determinantes sociais de saúde em camadas, do nível individual aos macrodeterminantes gerais. Os indivíduos estão na base com suas características de idade, sexo, genética. Em seguida, vem a camada do comportamento e dos estilos de vida, na qual se localiza a tensão entre arbítrio individual e condicionantes sociais. A camada sequente se refere às redes de apoio comunitário. O próximo nível representa os fatores relativos a condições de vida e trabalho, acesso a alimentos, habitação, serviços essenciais, saúde, educação. Na última camada, abordam-se os macrodeterminantes, econômicos, culturais e ambientais.

Buss & Pellegrini Filho admitem que estabelecer hierarquias e mediações de determinações entre fatores socioeconômicos e políticos e a saúde de indivíduos e grupos é desafiante. As relações de causa e efeito não são diretas, as diferenças de escala não se explicam mutuamente entre contextos diversos, e “*não basta somar os determinantes de saúde identificados em estudos com indivíduos para conhecer os determinantes de saúde no nível da sociedade*” ^1^ (p. 81). Buss & Pellegrini Filho ^1^ (p. 83) esclarecem que “*apesar da facilidade da visualização gráfica dos DSS* [determinantes sociais de saúde] *e sua distribuição em camadas, segundo seu nível de abrangência, o modelo não pretende explicar com detalhes as relações e mediações entre os diversos níveis e a gênese das iniquidades*”.

As observações de Buss & Pellegrini Filho ^1^ quanto ao que o modelo “não pretende explicar” permite questionar o modo de a epidemiologia dos fatores de risco operar de forma linear com variáveis binarizadas, tendendo a congelar e fragmentar processos e bloquear interações. Linearidade e binarização impedem o reconhecimento da interseccionalidade e interditam a construção de redes de relações e contextos históricos e sociais em movimento e transformação.

O não detalhamento e não esclarecimento de relações e mediações entre fatores, camadas, níveis do modelo descrito podem ser entendidos como próprios de pensamentos científicos que evitam ou não desejam o exame aprofundado, o que significa ocultar, invisibilizar. A não explicação dos contextos em que se estabelecem os determinantes sociais de saúde evidencia limitações epistêmicas importantes, e em contraposição se colocam formulações complexas da determinação social da saúde.

## Sobre determinantes e determinação social da saúde

A medida do biológico e do social no processo saúde-doença-cuidado passou a constituir a discussão do que sejam “determinantes sociais de saúde” e “determinação social da saúde”. Borghi et al. ^5^ (p. 884) entendem que tensionando-se o “*tema mediante a crítica epistemológica e filosófica*”, as denominações de “*determinação social do processo saúde-doença e determinantes sociais da saúde*” opõem “*um mosaico de concepções, com graus variados de convergência e divergência*”. Percebe-se, entretanto, que a ênfase na determinação social como crítica ao funcionalismo dominante na ideia dos determinantes é presente na teorização construída na América Latina.

Para Morales-Borrero et al. ^6^, as propostas dos determinantes sociais de saúde e da determinação social da saúde expressam diferentes referenciais “ético-políticos”. A primeira forma de pensar deriva da Epidemiologia Social anglo-saxônica, e a segunda vem da Medicina Social e da Saúde Coletiva latino-americanas e é fundamentada no materialismo histórico e teorias críticas das Ciências Sociais. A primeira visão considera a inserção social do indivíduo como geradora de vulnerabilidade e adoecimento, de acordo com a exposição diferencial resultante das iniquidades, mas não explica as relações de poder em tensão. A segunda perspectiva procura esclarecer as relações da determinação social da saúde com padrões de trabalho e consumo, falência dos suportes sociais, percepções culturais ligadas à insalubridade, contextos de classe social, gênero e etnia.

A ideia de que saúde e doença são socialmente inscritas implica em termos, conceitos, correlações que sustentam modelos representativos. Para Vieira-da-Silva & Almeida Filho ^7^ (p. S217-8) falta aprofundamento teórico às propostas explicativas dos determinantes sociais da saúde, estabelecendo-se uma “*confusão conceitual*” que resulta na não diferenciação entre “*inequidade e iniquidade*” e no tratamento das noções de “*equidade e igualdade*”, e seus contrários “*desigualdade, diferença e iniquidade*”, como sinônimos.

Almeida Filho ^8^ (p. 368) associa o tema de desigualdades sociais com justiça e admite que, por influência de países do Norte, uma unanimidade retórica simplificadora em torno do significado do termo “*equidade*” provoca contradições na consideração das relações com a saúde. No “*debate conceitual sobre determinantes em saúde, no Brasil e no mundo*”, apresenta-se uma “*quase unanimidade retórica em prol da equidade*” que “*impede averiguar a sinceridade política dos que formulam discursos politicamente referenciados*”. Muitos, “*até com a desculpa do interesse científico, muitas vezes apenas contemplam a persistência das iniquidades sociais no mundo*”. Almeida Filho adverte que “*há um grande perigo nessa retórica*”, o de “*deixar-nos desatentos e desarmados*” diante da “*possibilidade de se despolitizar a questão da saúde mediante a mera constatação distanciada da existência, quase naturalizada, de disparidades na ocorrência de doenças e eventos relativos à saúde*”.

Vieira-da-Silva & Almeida Filho ^7^ e Almeida Filho ^8^ enfatizam a conotação ética e política dada pelo epidemiologista equatoriano Jaime Breilh à diferenciação entre os conceitos de inequidade e iniquidade. Para Breilh ^9^, são as iniquidades que produzem desigualdade, injustiça, exclusão e opressão social. As iniquidades, potencializadas na associação tríplice de gênero, classe, raça/etnicidade, devem ser pensadas como evitáveis e perversas, dependentes da vontade política que as produz e mantém.

Breilh propõe uma dialética da determinação social da saúde que articula em movimento e transformação mútua níveis geral, particular e singular. Contingências pessoais e arbítrios individuais “*geram ou recriam condições particulares*” de socialização na ordem macro que reproduzem “*condições para o devir dos fenômenos de ordem microssocial*” ^9^ (p. 45). Saúde é objeto multidimensional, complexo, processo que “*se realiza na dimensão geral da sociedade, na dimensão particular dos grupos sociais e na dimensão singular dos indivíduos e seu cotidiano*” ^9^ (p. 43). “*Não é primordialmente individual-objetiva-contingente*”, nem tampouco “*coletiva-objetiva-determinada*”, mas é “*sempre e simultaneamente, o movimento de gênese e reprodução possibilitado pelo concurso de processos individuais e coletivos*” ^9^ (p. 45).

A dialética de Breilh enfatiza a inscrição intercultural da saúde. Para o autor ^9^ (p. 50), o predomínio da “*racionalidade eurocêntrica e androcêntrica*” e o “*uniculturalismo da ciência*” devem dar lugar a uma “*redescrição*” da relação entre o científico e o popular, constituindo uma “*negociação dos conhecimentos*” ^9^ (p. 56) entre pessoas, classes, etnias, gêneros.

Segundo Breilh ^9^, a categoria estilos de vida, funcional na epidemiologia dos fatores de risco como operador entre salubridade e insalubridade, apresenta-se nas teorias dominantes dos determinantes sociais da saúde de modo a denotar opção ou escolha e culpabilizar as vítimas pela insalubridade e adoecimento. Ainda segundo o autor, não somos expostos aos riscos, mas esses nos são impostos pela ordem social excludente.

A teoria do risco, centralizada nos estilos de vida, é, para Breilh ^9^ (p. 202), “*de enorme utilidade para os modelos de gestão neoliberal e para a manipulação da hegemonia na saúde*”, constituindo “*uma epidemiologia sem memória e sem sonhos de emancipação, presa à ditadura de um presente*” mantido à custa de “*mudanças cosméticas que deixam intacta a estrutura insalubre*”.

Examinando a “*colonização do saber epidemiológico*”, Sevalho ^10^ (p. 5631) sugere que a incorporação da dimensão social à “*epidemiologia dos fatores de risco operada pela ideia de estilo de vida, central na teoria dos determinantes sociais de saúde*”, reflete uma construção epistêmica conservadora, “*compondo a dominação pelo saber biomédico*” e evitando a vinculação desconfortável com a ordem política neoliberal.

Debate relevante sobre determinação social da saúde aconteceu com a publicação de artigo de Maria Cecília Minayo ^11^ questionando a referida categoria teórica. A discussão envolveu respostas de Almeida Filho e Breilh, autores expoentes do Sul que pensam a determinação social da saúde fora dos contextos norte-americano e eurocêntrico.

Almeida Filho ^12^ e Breilh ^13^ respondem a Minayo com respeito, porém com veemência, apontando inadequações e apagamentos no tratamento dado pela autora à reflexão crítica latino-americana sobre o tema. Para Almeida Filho ^12^ (p. 1), a provocação de Minayo “*confunde o princípio filosófico do determinismo, a categoria determinação e a noção de determinante*”. Ao se contrapor ao cancelamento do debate sobre a determinação social da saúde, Almeida Filho chama a atenção para a relevância do trabalho de Breilh. Por sua vez, Breilh ^13^ lamenta que Minayo questione a validade de tema tão necessário ao atual contexto político mundial.

Breilh ^13^, respondendo a Minayo, pontua que a determinação social da saúde é elaboração teórica fundamental na produção de uma ciência crítica politicamente relevante. Complexa, a determinação social da saúde envolve a geração de processos em múltiplas dimensões de interdependência e autonomia, e incorpora a contradição de processos saudáveis e insalubres e “*outras formas de movimento*” da vida social, dos ecossistemas e da saúde humana, como “*causação*”, “*retroalimentação*”, “*estocasticidade*”, “*incerteza*” ^13^ (p. 2).

No debate citado, Almeida Filho ^12^ (p. 3) salienta uma “*sobredeterminação*”, caracterizada como “*formas particulares de sínteses de muitas determinações*”. O autor indica a origem do termo sobredeterminação em Marx e cita que sua implicação em saúde foi estabelecida por Freud ao lidar com cadeias articuladas de elementos causadores das neuroses e outros envolvendo o inconsciente. Na interpretação freudiana, a sobredeterminação relaciona elementos que, sem ter necessariamente estreita mutualidade, ganham expressão quando se entrelaçam. Segundo Almeida Filho, Lacan recupera o sentido freudiano da sobredeterminação na formação do inconsciente como um conceito referente à “*elementos de reduzido valor psíquico*” que ganham força quando se juntam e convergem na criação de “*vetores novos e mutantes de produção de efeitos*” ^12^ (p. 2).

Almeida Filho ^12^ atribui a Luis David Castiel, epidemiologista que, como ele, tem trânsito significativo na Saúde Mental, o relacionamento da sobredeterminação freudiana com a Saúde Coletiva. Coutinho et al. ^14^ apontam a necessidade de a Epidemiologia buscar referências epistemológicas que ultrapassem a fixação na prevenção e a libertem da dependência das categorias causalidade, predição e determinação. Uma forma de alcançar esse propósito, segundo os autores, é explorar a articulação dos conceitos de sobredeterminação, como múltiplas determinações em rede, e contingência, como acontecimento possível que prescinde de necessidade, avançando na construção de bases epidemiológicas teórico-metodológicas complexas e não determinísticas, distintas da causalidade de lógica linear e da predição mecânica.

Articular sobredeterminação e contingência, como propõem Coutinho et al. ^14^, implica na consideração da complexidade e da imprevisibilidade, e no consequente rompimento com a visão de uma temporalidade espacializada, conformada como ordem linear de sucessão, que prevalece no raciocínio epidemiológico causalista.

## Determinação social da saúde e complexidade epistêmica na abordagem da pandemia de COVID-19

Ilya Prigogine ^15^ ^,^ ^16^ e Edgar Morin ^17^ ^,^ ^18^ são pensadores fundamentais das epistemologias da complexidade. Aliam ser humano e natureza e percebem sistemas sociais e naturais abertos, em movimento e transformação, trocando matéria, energia e informação com o exterior, regidos segundo lógicas não lineares e não deterministas, orientados pela irreversibilidade temporal e assimetria entre passado e futuro, associados a instabilidades, flutuações e bifurcações.

O sentido epistemológico da complexidade se evidencia nas reflexões de Almeida Filho ^19^ e de Breilh ^20^ ^,^ ^21^ ^,^ ^22^ sobre a determinação social da pandemia de COVID-19 nas sociedades do Sul.

Almeida Filho propõe uma modelagem complexa não quantitativa da pandemia de COVID-19. O autor a entende como objeto que não se restringe à emergência de um patógeno, nem aos elementos clínicos da nova entidade, nem aos processos de disseminação, nem aos indicadores epidemiológicos de morbimortalidade, nem à “*infodemia de fake news, mitos e mentiras*”, nem ao medo ou contingências econômicas e políticas que a acompanham. A pandemia implica uma “*totalidade singular*” que unifica todos estes aspectos “*por meio de uma integralização heurística*” que não fragmenta a análise em “*medições, mediações, descrições, efeitos, correlações e narrativas*” ^19^ (p. 97).

Identificando uma hegemonia epistemológica colonizadora na epidemiologia, Almeida Filho ^19^ fundamenta sua proposta no conhecimento produzido no Sul global, e busca referência na Epistemologia da Complexidade do argentino Juan Samaja. O modelo de inspiração samajiana, elaborado por Almeida Filho ^19^ (p. 103), segue uma “*multiplanidade*”, relacionando a pandemia a uma “*sobredeterminação ocorrendo simultaneamente e recursivamente nos diversos planos da vida humana*”, envolvendo interfaces de sistemas biológicos, ecossociais, socioculturais. “*Processos patológicos, estados de saúde e correlatos*” ^19^ (p. 103) se constituem como “*holopatogênese*”, resultante da “*tensão entre agressores e defesas corporais*” ^19^ (p. 106) arranjados em complexas tramas de determinação. Segundo Almeida Filho, o conceito se adequa aos fenômenos relativos à saúde-doença, percebidos como objetos plurais, polissêmicos, que requerem abordagem por meio da síntese.

Almeida Filho critica a valorização exclusiva da ordem biodemográfica, que aponta como limitação característica das disciplinas da saúde. Em contraposição, o autor ^19^ (p. 103) enreda uma dimensão “*microestrutural*”, de correspondência “*molecular ou celular*”, passando pelos níveis clínicos, individuais, e epidemiológicos, com dimensões ecossistêmicas e simbólicas da ordem cultural. “*Nas pandemias e outros eventos epidemiológicos críticos*”, esclarece Almeida Filho ^19^ (p. 105), a ordem simbólica sobredetermina “*conjuntos, subespaços e planos de emergência dos complexos fenomênicos da saúde-enfermidade-cuidado*”.

Segundo Almeida Filho ^19^ (p. 104), “*prioridade especial deve ser concedida ao conceito de vulnerabilidade e seus correlatos, particularmente eficaz na dimensão social e na esfera subjetiva*”. Em encontro de pós-graduação realizado no Instituto de Saúde Coletiva da Universidade Federal da Bahia, tematizado como *Ciência, Saúde e Complexidade*, Almeida Filho ^23^ reafirma que não há razão para cancelar o debate sobre “*determinantes sociais*” e “*determinação social*”, e propõe usar o conceito de risco na perspectiva dos determinantes e o de vulnerabilidade no contexto da determinação social. “*Risco é determinante, vulnerabilidade é determinação*”, diz Almeida Filho no evento.

Vulnerabilidade, conforme Ayres et al. ^24^, abrange risco e formas individuais e coletivas de seu enfrentamento. É conceito cujo lastro complexo e implicação sociopolítica transcendem o referencial probabilístico do risco.

Segundo Breilh ^20^, os modelos positivistas, cartesianos, de explicação do processo saúde-doença fragmentam o mundo em partes estáticas, separadas, e as associam de forma linear, bloqueando a percepção de contexto social. Limitam-se os modelos a descrever e calcular probabilidades de fenômenos sem explicar ou comprometer “*a sociedade e sua estrutura de poder*” ^20^ (p. 45).

Breilh desenvolve um projeto emancipador, não funcional ao poder. O autor ^20^ (p. 72) percebe a “*intrínseca conformação contraditória*” que dá à saúde a dimensão de movimento que arranja processos saudáveis, protetores e processos destrutivos. A pandemia de COVID-19 é ancorada em uma “*matriz de processos críticos com base social e territorial*” de entendimento dos modos de vida típicos que se reproduzem segundo: “*modos e padrões de trabalho*”, “*modos e padrões de consumo”*, “*modos/capacidade de organização e suporte social*”, “*modos/capacidade de construção de identidade cultural própria*” e “*modos de relação com o ecossistema*” ^20^ (p. 73). Para Breilh ^20^ (p. 73-4), “*a tipologia dos modos de vida permite caracterizar o grau de consolidação saudável ou insalubre de coletividades no território*” em correspondência com “*padrões de exposição e vulnerabilidade*”.

Breilh ^20^ ^,^ ^21^ ^,^ ^22^ interpreta a pandemia segundo sua dialética da determinação social da saúde. Na dimensão geral, definem-se “*a reprodução social e as relações metabólicas ambientais mais amplas de natureza-sociedade*”; no nível particular, movimentam-se os modos de vida dos coletivos “*com suas relações metabólicas sociais e específicas (quer dizer, classe social, gênero e poder etnocultural)*” e entre “*modos de vida*” e “*estilos de vida*” se estabelece uma reciprocidade entre arbítrio e condicionamento social, e no nível individual expressam-se as “*pessoas/famílias com seus estilos pessoais específicos de vida e constituições psicológicas e corporais (quer dizer, fenotípicas, genotípicas, psicológicas e espirituais)*” ^20^ (p. 74).

A dialética de Breilh ^22^ (p. 29) “*não reconhece nenhum progresso ou desenvolvimento linear da sociedade e sua saúde, nem tampouco sustenta a primazia absoluta do material econômico sobre o cultural-político, nem do social sobre o biológico-natural*”. O entrelaçamento da base material com as expressões sociopolíticas e socioculturais é um ir e vir, “*entre subsunção e autonomia*”, tal como acontece com as relações “*individual-coletivo*” e “*sociedade-natureza*”.

O “*metabolismo sociedade-natureza*” relaciona historicamente uma base natural e uma natureza socialmente transformada. A “*subsunção-autonomia-relativa*”, que Breilh ^22^ (p. 29) revela ser inspirada na epistemologia de Juan Samaja, é chave para “*suprimir a predominância do causalismo empírico*”. É a qualidade da reprodução social pela qual um subsistema mais complexo se impõe a outro menos complexo, que tende, por sua vez, a expressar autonomia relativa.

Breilh ^20^ ^,^ ^21^ ^,^ ^22^ considera uma quarta revolução industrial, surgida como convergência social de tecnologias e sistemas cibernéticos e biológicos. A pandemia de COVID-19 não pode ser compreendida, e tampouco podem ser estabelecidas estratégias efetivas de enfrentamento, se não é considerada a agressividade do capitalismo da quarta revolução industrial, que avança acelerado, livre de regulação ^21^ ^,^ ^25^.

A quarta revolução industrial, concepção do economista neoliberal Klaus Schwab ^26^, sucede a primeira (da invenção da máquina a vapor), a segunda (da eletricidade), a terceira (da informática), e se refere à coexistência e conexão de descobertas como física quântica, inteligência artificial, robótica, nanotecnologia, sequenciamento genético, engenharia genética, comunicação via internet, novas formas de armazenamento de energia. Como fenômeno social, a quarta revolução industrial se caracteriza pela convergência de velocidade, flexibilidade, profundidade, intensidade, alcance, impacto sistêmico, podendo ter efeitos prejudiciais ainda desconhecidos sobre comportamento e relacionamento humanos, conforme Schwab admite.

Pensando a determinação social da pandemia de COVID-19, Breilh ^21^ (p. 12) usa a metáfora bíblica dos quatro cavaleiros do apocalipse, peste, morte, fome e guerra, para definir os “*quatro cavaleiros do apocalipse da saúde*”, quatro “*ameaças globais unidas por uma matriz social-civilizatória*”: eclosão de um ciclo de pandemias no século XXI, eclosão e aceleração de mudanças climáticas catastróficas, crescente reprodução da desigualdade social nas cidades neoliberais e na nova ruralidade injusta em expansão, e “*o vírus da desinformação (infodemia real)*”.

A quarta revolução industrial em curso dissemina e acelera a iniquidade em saúde, e tem como consequência a instauração de condições sociais e ecossistêmicas favoráveis aos ciclos pandêmicos ^22^. A ciber-determinação social da vida e da saúde, segundo Breilh ^21^, é concomitante e inter-relacionada com formas opressoras de controle social direto que ainda persistem.

## Determinação social da saúde, colonialidade, longa duração

A multiplanidade/sobredeterminação de Almeida Filho e a dialética da determinação social da saúde de Breilh são construções epistemológicas interdisciplinares críticas alternativas ao conhecimento hegemônico de fundamentação europeia e norte-americana. Guardada a originalidade de cada proposta, Almeida Filho e Breilh, com sua perspectiva epistemológica complexa, seu compromisso com a transformação social e seu sentido descolonizador, inspiram a aproximação entre determinação social da saúde e a discussão decolonial. Na interdisciplinaridade, é estabelecida uma negociação de saberes que envolve visões de mundo com pontos críticos em comum.

Exploramos essa proximidade tendo como referência o tempo enquanto elaboração epistemológica. Articulamos as leituras de Braudel e Wallerstein sobre a longa duração, o sistema mundo e a formação histórica do capitalismo ao pensamento decolonial latino-americano, incorporando o conceito de colonialidade à teoria crítica da determinação social da saúde. Consideramos, então, a postulação de Mignolo ^27^ (p. 2) de que o capitalismo não poderia existir sem a colonialidade, “*o lado mais escuro da modernidade*”.

Fernand Braudel é o grande historiador da segunda geração da historiografia dos *Annales*, constituída por autores movimentando-se em torno da revista francesa *Annales D’Histoire Économique et Sociale*, fundada em 1929 por Lucien Febvre e Marc Bloch. Como esclarece José Carlos Reis, historiador brasileiro estudioso do tema, a nouvelle histoire dos Annales une-se às ciências sociais tendo como fundamento um tempo que não se subordina nem à exclusividade do acontecimento nem das estruturas. Incorporando a simultaneidade sem renunciar à sucessão, “*cria-se uma permanência sobre a qual se articulam mudanças mais ou menos lentas*”, diz Reis ^28^ (p. 20). Sensível à resistência da objetividade diante da subjetividade, o tempo criado é complexo, multidirecionado, descontínuo, assimétrico, indeterminado, composto de ritmos e irreversibilidades particulares plurais.

Entendendo que todos têm direito à história, os historiadores da nova história mergulharam na estrutura, na longa duração, na qual estão imersos os seres humanos comuns, anônimos, sem perder de vista o acontecimento, o tempo curto. Perceberam que é na profundidade, escura e pouco visitada, da história que estão a lentidão da cultura, a resistência dos hábitos e dos valores, os movimentos repetitivos, por vezes inconscientes, que caracterizam a cotidianidade. Essa historiografia fez surgir personagens esquecidos, invisibilizados, como as mulheres, os pobres, os marginais, e temas de investigação não explorados emergiram, como os sentidos, sonhos, costumes, mentalidades, de forma que novas abordagens passaram a utilizar novas fontes documentais. Elementos produzidos involuntariamente tornaram-se fontes e a história não se esgota mais nos documentos oficiais, próprios de uma história de Estado, em que a historiografia tradicional só percebia o evento, o acontecimento rápido, e identificava vultos, heróis, datações pontuais.

A proposta de Braudel ^29^ é contraposta à história tradicional, acontecimental, que privilegia a narrativa de pouco fôlego. Para Braudel ^29^ (p. 265), “*o tempo curto é a mais caprichosa, a mais enganadora das durações*”. Braudel se refere aos “*tempos múltiplos e contraditórios da vida dos homens*” ^29^ (p. 262), à “*oposição viva, íntima, repetida indefinidamente entre o instante e o tempo lento a escoar-se*” ^29^ (p. 263). O historiador envolve os objetos de investigação na multiplicidade das durações, em uma rede de tempos diferenciados, não reduzida nem à longa, nem à média, nem à curta duração. Braudel pensa um tempo coletivo, não linear, complexo, interdisciplinar, que não tem a duração do indivíduo, mas de décadas, séculos. Acontecimentos, conjunturas e estruturas concorrem entre o deixar-se levar pela rotina e a decisão, com a participação do inconsciente e da intencionalidade no caminhar da vida. Passado e presente se contaminam, não existe ruptura absoluta entre eles.

Braudel ^30^ contextualiza a formação histórica do capitalismo nessa dialética da duração, relacionando vida material, economia de mercado, economia capitalista. As trocas rápidas, no limite dos burgos, os camelôs, as lojas, os mascates, passando pelas feiras, a economia de mercado, as operações à distância, entre cidades, regiões, a participação de intermediários, as bolsas, o crédito, os sistemas bancários com suas transações internacionais, o livre comércio, os monopólios, a coexistência das economias-mundo. As economias-mundo de Braudel ocupam um espaço dado, aceitando um polo e zonas sucessivas de participação hierarquizada. Desde os sistemas de trocas, a palavra que vem ao espírito de Braudel, na observação do tempo longo, é “capitalismo”. O autor diz empurrá-la porta afora quando a vê surgir, mas a palavra retorna, sem substituto viável.

O trabalho, a precariedade da vida, a pobreza, as guerras, a fome, as epidemias enredam-se na complexidade da história. Como diz Braudel ^30^ (p. 16), na longa duração assiste-se ao “*longo cortejo das doenças*”, que “*explode por combinação de espécies microbianas*” depois da descoberta da América.

Em meados das décadas de 1930 e 1940, Braudel esteve no Brasil durante a estruturação da Universidade de São Paulo. O historiador ilustrava sua ideia da duração citando um enxame de vagalumes que lhe foi trazido por uma noite numa pequena cidade da Bahia: as luzes dos vagalumes piscando seriam os acontecimentos, só percebidos contra o fundo da noite, a escuridão do tempo longo. Em 1985, no encontro de Châteauvallon, que o homenageou pouco antes de morrer, Braudel ^31^ afirmou que os mais belos anos de sua vida os passou no Brasil.

Outro destacado pensador do tempo longo é o sociólogo e historiador norte-americano Immanuel Wallerstein ^32^ ^,^ ^33^, com sua teorização do “capitalismo histórico” e sua análise do “sistema mundo capitalista”.

A unidade de análise de Wallerstein ^32^ são os sistemas históricos, com três modalidades em coexistência, classificadas segundo forma e lógica de reprodução social. Os “minissistemas”, formações de tempo curto em que prevalecem trocas em pequena escala regidas pela reciprocidade, com padrões culturais e de governo homogêneos; os “impérios mundiais”, estruturas políticas vastas, sistematizadas por meio da extração de tributos, que abrigam grande diversidade de padrões culturais; as “*economias mundiais*”, grandes e “*desiguais cadeias de estruturas de produção, dissecadas por múltiplas estruturas políticas*” ^32^ (p. 459), com lógica baseada na distribuição desigual do excedente acumulado em favor daqueles capazes de realizar monopólios. Criam-se e recriam-se minissistemas e economias-mundo, absorvidos ou destruídos dentro dos impérios mundiais, até o surgimento do “sistema mundial moderno”, desde a expansão de uma única economia mundial europeia a partir do século XVI, segundo Wallerstein. O capitalismo expandiu-se “*absorvendo nesse processo todos os minissistemas e impérios mundiais existentes*”, e no século XX “*existia pela primeira vez apenas um sistema histórico sobre o globo*” ^32^ (p. 460).

A autoexpansão, entendida por Wallerstein como força do capitalismo, deve ser compreendida em sentido físico e discursivo como dinâmica estrutural que se desenvolve na longa duração do tempo contando com a promoção mútua de seus ingredientes fundamentais, que o autor chama de “*o trio universalista da ciência, dos direitos humanos e da meritocracia*” ^33^ (p. 111). Esses elementos potencializam-se miticamente enquanto construção cultural. Para Wallerstein, o “universalismo” é o que sustenta ideologicamente o capitalismo histórico. É “uma epistemologia” e “uma fé” que essencializam afirmações gerais e representam verdades permanentes sobre o mundo físico e o mundo social, cabendo à ciência revelá-las para eliminar as subjetividades.

À semelhança da dialética da duração de Braudel, em que mudanças são visíveis como acontecimentos de tempo curto quando emergem da lentidão das permanências, para Wallerstein ^32^ (p. 466), “*o que a análise dos sistemas mundiais requer é uma avaliação da centralidade desses pretensos ‘acontecimentos-chaves’ em termos da longa durée do sistema histórico no qual eles ocorreram*”.

Na argumentação de Wallerstein ^33^, o racismo e o sexismo são importantes na construção do capitalismo histórico, na medida em que estabelecem hierarquizações sociais que alimentam o sistema no que tem de perverso. Juntos, sexismo, que relega a mulher ao trabalho não produtivo, e racismo, como dispositivo de estratificação da força de trabalho, conformam “*uma estrutura de humilhação opressiva*” ^33^ (p. 88). “*Tanto o sexismo como o racismo são processos sociais em que a ‘biologia’ define posições*” ^33^ (p. 89), justifica a desigualdade e limita a expectativa de uma vida melhor.

À opressão da precariedade da vida e da discriminação injuriosa, unem-se doenças e epidemias. Wallerstein ^33^ faz um balanço equilibrado da saúde, considerando as melhorias históricas da saúde pública. Pontua, no entanto, “*as consequências devastadoras do cruzamento de estoques genéticos de organismos parasitários, oriundo do avanço tecnológico dos transportes*” que integra a “*expansão da economia capitalista*” ^33^ (p. 101). O processo dizimou mais de um terço da população das Américas, e semelhante tragédia ocorreu “*nas zonas mais remotas da África, Ásia e Europa*” ^33^ (p. 101). O autor identifica, ainda, a difusão de doenças causadas pelos desgastes ambientais e o risco do surgimento de outras relacionadas à acumulação capitalista.

Braudel e Wallerstein dedicaram décadas a suas pesquisas. Braudel sintetizou a dialética da duração sob a marcação da longue durée e criou a ideia das economias-mundo. Wallerstein deu tonicidade política à hierarquização social, ao racismo e ao sexismo e conectou esses dispositivos na formação do sistema mundo moderno, capitalista, no tempo longo.

Sobre Wallerstein, Braudel ^30^ (p. 70) diz que “*os nossos pontos de vista, quanto ao essencial, são idênticos*”. Ele e Wallerstein ^34^, entretanto, reconhecem alguma divergência sobre a coexistência de múltiplas economias-mundo.

A leitura do capitalismo como estrutura histórica movimentando-se entre centro e periferia foi, segundo o sociólogo peruano Aníbal Quijano, autor seminal do pensamento decolonial, reelaborada por Wallerstein como sistema mundo moderno, desde a confluência entre a “*visão marxiana do capitalismo como um sistema mundial e a braudeliana sobre a longa duração histórica*” ^35^ (p. 95).

A crítica decolonial identifica o apagamento da história, de existências, das culturas e dos conhecimentos da América Latina, ancorado na invenção de um tempo linear evolutivo fundamentado na atribuição de uma primordialidade universal à modernidade ocidental, europeia, entendida como marco civilizatório. O giro decolonial descortina a ancestralidade latino-americana como lócus das experiências que forjaram historicamente a opressão capitalista no tempo longo. O pensamento decolonial releva o econômico e o cultural, recusando economicismos e culturalismos extremos, e, embora inspirado em aspectos dos estudos culturais e pós-coloniais, é crítico da vinculação eurocêntrica dessas abordagens descoloniais.

A associação do norte-americano Wallerstein com o pensamento decolonial latino-americano é identificada por Ballestrin ^36^. Os autores decoloniais Santiago Castro-Gómez e Ramón Grosfoguel informam do trabalho conjunto desenvolvido na Universidade do Estado de Nova Iorque (Estados Unidos), na década de 1990, por Quijano e Wallerstein, quando este último era diretor do Centro Fernand Braudel, de Paris (França) ^37^. Desde os anos 1970, Wallerstein pensava o capitalismo histórico e a “análise do sistema-mundo” e Quijano se projetava inicialmente com a “teoria da dependência” latino-americana e posteriormente com a criação do conceito de colonialidade ^37^.

A colaboração entre Quijano & Wallerstein ^38^ (p. 585) originou publicação conjunta na qual consideram uma “*etnicidade*”, sustentada por “*um consciente e sistemático racismo*”, concebida na expansão da “*americanidade*” no contexto da colonialidade no sistema mundo moderno. Para Quijano & Wallerstein, em países periféricos como o Brasil, a segregação explícita é ocultada nas dobras de uma hierarquização étnica apoiada em um pareamento entre universalismo e meritocracia, que possibilita dissimular o racismo e sistematizar cultural e politicamente privilégios dos “*estratos étnicos dominantes*” ^38^ (p. 586).

Segundo Castro-Gómez & Grosfoguel ^37^, nos anos 1990 e início dos 2000, do diálogo entre a colonialidade de Quijano e o sistema-mundo do braudeliano Wallerstein, desenvolveram-se discussões, publicações e eventos nas universidades norte-americanas do estado de Nova Iorque, Duke, da Carolina do Norte, e da Califórnia, em Berkeley, e nas latino-americanas Universidade Central da Venezuela, Universidade Javeriana de Bogotá (Colômbia) e Universidade Andina Simón Bolívar do Equador. As atividades reuniram pensadores latino-americanos - como os argentinos Walter Mignolo e Enrique Dussel, os venezuelanos Edgardo Lander e Fernando Coronil, os porto-riquenhos Nelson Maldonado-Torres e Ramón Grosfoguel, o colombiano Santiago Castro-Gómez, a norte-americana radicada no Equador Catherine Walsh - e envolveram a participação de feministas e pós-coloniais - como a cubana Sylvia Wynter e a indiana Chandra Mohanty. Na primeira década do século XXI, fortaleceu-se a personalidade organizacional do Grupo Modernidade/Colonialidade, intensificaram-se as colaborações com intelectuais como o jamaicano Lewis Gordon, estudioso divulgador do pensamento do psiquiatra martinicano Frantz Fanon, e o português Boaventura de Sousa Santos, e consolidou-se o pensamento/movimento decolonial como epistemologia crítica.

A designação “*decolonial*” se deve a Catherine Walsh ^39^ (p. 24-5), para quem a supressão da letra “*s*” da descolonialidade e do descolonial, longe de promover um anglicismo, é tanto “*jogo linguístico*” como “*caminho de luta contínuo*” que “*pretende marcar uma distinção com o significado em castellano do ‘des’*”, definindo a passagem do colonial para o não colonial. Walsh aponta o uso do termo pelo projeto modernidade/colonialidade desde 2004, e esclarece que a nomeação decolonial quer evidenciar “*posturas, posicionamentos, horizontes e projetos de resistir, transgredir, intervir, in-surgir, criar e incidir*”, para “*identificar, visibilizar e alentar ‘lugares’ de exterioridade e construções alter-(n)ativas*” ^39^ (p. 25).

O conceito de colonialidade, criado por Quijano ^40^ ^,^ ^41^, é fundamento do pensamento decolonial, e se distingue do colonialismo referente à vigência administrativa dos governos coloniais. Colonialidade vai além de colonialismo, em termos temporais e epistêmicos.

Segundo Quijano ^40^ ^,^ ^41^, a dominação europeia das Américas é instituída por meio da criação de hierarquizações econômicas, culturais, étnicas, de gênero, amparadas em dispositivos de controle do processo de trabalho e meios de produção e na invenção da categoria mental de raça, promovida como característica biológica justificadora e avalizadora da escravidão, da servidão, da exploração de negros e indígenas, não europeus, não brancos, não cristãos. Sobre esses alicerces, Quijano pensa uma “colonialidade do poder” e “uma colonialidade do saber”.

Mignolo ^27^ e Maldonado-Torres ^42^ dão sequência à elaboração de Quijano e conformam a “colonialidade do ser”, e Walsh ^43^ acrescenta uma “colonialidade da Mãe Natureza”. Para Maldonado-Torres ^42^, a colonialidade do poder se refere à exploração e à dominação, a colonialidade do saber diz respeito à epistemologia e à produção de conhecimento, e a colonialidade do ser diz da experiência vivida da colonialidade, suas interfaces de subjetividade e intersubjetividade e o impacto na linguagem. A proposta de Walsh ^43^ (p. 138) confronta a “*divisão binária natureza/sociedade*” que descarta o “*mágico-espiritual-social*” que sustenta “*os sistemas integrais*”, ancestrais, do conhecimento e da vida.

Para Quijano ^35^ (p. 116), as regras de classificação e dominação capitalista que definem a colonialidade articulam-se “*dentro de um padrão social de poder de longa duração*”. Conforme Castro-Gómez & Grosfoguel, a discussão decolonial revela que as “*exclusões provocadas pelas hierarquias epistêmicas, espirituais, raciais/étnicas e de gênero*” ^37^ (p. 14) são elementos de “*longa duração*” implantados pela modernidade ocidental “*durante os séculos XVI e XVII*”, integrados à “*dominação e exploração econômica do Norte sobre o Sul*” como “*estrutura etno-racial de longa duração*” ^37^ (p. 17). O capitalismo, segundo Castro-Gómez & Grosfoguel ^37^, não é sistema restrito ao econômico ou ao cultural, ou hierarquia fechada na qual prevalecem lógicas autônomas sobre as demais. É uma “heterarquia”, na qual a dominação é formação econômica-política-cultural de “*processos complexos, heterogêneos e múltiplos, com diferentes temporalidades, dentro de um só sistema-mundo de longa duração*” ^37^ (p. 18), em que se articulam poder e opressão.

Conjugando a dialética da duração de Braudel e as concepções de capitalismo histórico e de sistema-mundo de Wallerstein, o pensamento decolonial insere a colonialidade na longa duração. É como elemento da longa duração que a colonialidade compõe a determinação social da saúde, seja inscrevendo-se socialmente como dispositivo gerador de insalubridade e sofrimento, produzindo e reproduzindo iniquidades econômicas, de gênero, étnicas, racismo, seja como construção epistemológica que invisibiliza culturas, promove epistemicídios, seja como colonialidade do saber que orienta a conceituação epidemiológica.

## Considerações finais

Wallerstein pensa sobre as crises do capitalismo e confessa incerteza sobre o que virá. Sua análise dos sistemas mundiais “*nasceu como protesto moral e, em seu sentido mais amplo, político*” ^32^ (p. 447), e constitui debate paradigmático contra o conservadorismo de uma historiografia que se conforma à visão linear de “*uma crença no triunfo inevitável do bem*” ^32^ (p. 470). No caminhar dos sistemas históricos, afirma Wallerstein, “*o determinado não é o simples, mas o complexo ou, na verdade, o hipercomplexo*” ^32^ (p. 469).

O avanço do capitalismo, para Breilh ^22^, cria, no século XXI, um movimento destrutivo de grandes proporções. A ordem neoliberal impõe o *“monopólio global de recursos agrícolas, industriais, financeiros e, mais recentemente, digitais*”, que produz “*uma regressão sistemática de direitos humanos, sociais e ambientais*” ^22^ (p. 44). A dominação é potencializada por “*mecanismos político-ideológicos, culturais e comunicativos*” ^22^ (p. 50) que disciplinam mentes, afetam a intimidade cotidiana e alienam as coletividades de suas necessidades estratégicas. O megaextrativismo capitalista físico e virtual tem impacto nefasto na determinação social da saúde.

O mundo é atravessado por migrações e diásporas forçadas pelo jogo neoliberal. No tempo longo, o ser humano é obrigado a, antes de viver, sobreviver. Migrantes fogem da opressão e, quando não perecem no trajeto, batem às portas de uma Europa que, armada do preconceito, não os quer, quando muito os tolera nos circuitos marginais. É imperativo que os migrantes se traduzam para serem incluídos nos ambientes sociais a que chegam, e esse processo de tradução cultural, como percebem os pensadores pós-coloniais Homi Bhabha ^44^ e Stuart Hall ^45^, são tensos, sempre próximos da perda de identidade e da marginalidade. A tradução cultural não permite alteridade pura; o resultado é hibridização que pode levar à resistência ou à alienação e domesticação. A colonialidade é iníqua, perversa, indigna.

No Brasil, uma reflexão pioneira sobre a determinação social da saúde sob o olhar decolonial foi realizada por Elis Mina Seraya Borde ^46^, e uma conclusão da autora é que a ausência da discussão sobre a migração é característica da visão hegemônica oficial. Fundamentada em documentos e bibliografia crítica, a autora pensou as abordagens das iniquidades étnico-raciais no contexto das interpretações dominantes da determinação social da saúde, tendo como referência o contraponto entre o discurso oficial e a “*perspectiva da Medicina Social e Saúde Coletiva Latinoamericana (MS-SC*) *e da ‘inflexão descolonial’ do Grupo Modernidade/Colonialidade (Grupo M/C)*” ^46^ (p. 9). Borde percebe um olhar abstrato da parte dos organismos que definem políticas públicas relativas à determinação social da saúde, ao lidar com a questão do racismo sem chegar a propostas efetivas de intervenção para transformação social. Incompletude teórica, dificuldade em afirmar o social, fuga de temas como migração e de termos como raça marcam os documentos estudados. Ao final, a autora esclarece sua proposta de “*quebrar os silêncios impostos pelo modelo de racionalidade*” dominante e explicitar a invisibilização da “*subalternização das populações não brancas e o racismo no sistema-mundo capitalista/colonial*” ^46^ (p. 127).

A epidemiologia dos fatores de risco isola fenômenos e esvazia o conteúdo sociopolítico do processo saúde-doença-cuidado. Prevalece a consideração de sistemas fechados, e a linearidade e o foco em fatores fragmentam processos e invisibilizam contextos históricos. Os padrões de salubridade são remetidos aos estilos de vida modelados pela cultura ocidental hegemônica e seu elogio da competitividade individualista. A objetivação privilegia variáveis binarizadas, como renda, sexo, cor da pele, em detrimento da complexidade da classe social, gênero e sexualidade, etnia, que melhor evidenciam e narram relações de hibridização, interseccionalidade, hierarquização e dominação. A integralidade ser humano-natureza é negada, o conhecimento biomédico ocidental eurocêntrico é universalizado; já os conhecimentos locais, saberes populares, são desconsiderados.

Para Castro-Gómez & Grosfoguel ^37^ (p. 17), é necessário pensar “*novos conceitos*” e “*nova linguagem*” para lidar com “*a complexidade das hierarquias de gênero, raça, classe, sexualidade, conhecimento e espiritualidade dentro dos processos geopolíticos, geoculturais e geoeconômicos do sistema-mundo*”. Atentos à recomendação, devemos explorar bases conceituais para a teoria crítica da determinação social da saúde.

Como aponta Prigogine ^47^ (p. 232), “*tempo e complexidade são conceitos que intrinsecamente apresentam relações mútuas*”. A integração do tempo, como fundamento epistemológico, ao pensamento crítico sobre a determinação social da saúde contribui para identificar a colonização do saber, revelar apagamentos, (re)constituir contextos históricos e durações cuja complexidade escapa ao funcionalismo cartesiano da epidemiologia dos fatores de risco, com sua visão fragmentada e congelada dos determinantes.

A lógica causal linear protege do não determinismo e da incerteza uma epidemiologia sem ambições de transformação social, acomodada a um mundo sem história. Com sua relação mudança-permanência, suas assimetrias, múltiplas temporalidades e irreversibilidades, e as buscas de fontes alternativas de informações e de novos objetos de investigação que a compõem, a duração braudeliana pode constituir aporte epistêmico valioso ao ser colocada ao lado de modelos complexos críticos como o da sobredeterminação de Almeida Filho e o da dialética da determinação social de Breilh, que privilegiam não linearidade, movimento, transformação.

A longa duração de Braudel, o sistema-mundo de Wallerstein e o conceito de colonialidade do pensamento decolonial expõem a determinação social da saúde à luz da crítica epistemológica, e resgatam da invisibilidade, da escuridão das prisões da longa duração nas quais estão imersos, aqueles que Frantz Fanon ^48^ chama de “os condenados da terra”, e os da saúde coletiva chamamos de vulnerabilizados.
